# Growing into gender: Funerary practice of boys and girls in the Bronze Age Maros culture (2100 − 1700 BC)

**DOI:** 10.1007/s12520-026-02578-3

**Published:** 2026-10-10

**Authors:** Mina Amzirkov, Marko Porčić, Katharina Rebay-Salisbury, Fabian Kanz, Michael Wolf, Marija Krečković Gavrilović, Sofija Stefanović

**Affiliations:** 1 Laboratory of Bioarchaeology, Department of Archaeology, Faculty of Philosophy, University of Belgrade, Belgrade, Serbia; 2 Department of Prehistoric and Historical Archaeology, University of Vienna, Vienna, Austria; 3 Austrian Archaeological Institute, Austrian Academy of Sciences, Vienna, Austria; 4 Unit of Forensic Anthropology, Medical University of Vienna, Vienna, Austria; 5 Department of Analytical Chemistry, University of Vienna, Vienna, Austria

**Keywords:** Amelogenin peptides, Sex, Gender, Bronze Age, Mortuary practices

## Abstract

This study investigates the relationship between biological sex and gendered funerary practices among children of the Bronze Age Maros culture in northern Serbia. Biological sex was determined for 77 non adult individuals (< 18–22 years), for 76 of them using peptide based sex identification from dental enamel, and for one individual previously published aDNA results were used (Žegarac et al. 2021). The results were compared with each individual’s body orientation and position, key elements of the Maros funerary norm known to reflect binary gender differentiation among adults. Statistical analyses revealed a strong association between peptide-determined biological sex and side of the body position, as well as a correlation between biological sex and body orientation. At Mokrin, biological sex was strongly associated with orientation of the body and moderately associated with body position. At Ostojićevo, the relationship was even stronger, but in a smaller sample. These findings confirm that boys were buried according to the male (north-south orientation, positioned on their left side) and girls according to the female norm (south-north orientation, positioned on their right side), mirroring the adult burials. The consistency of these practices demonstrates that binary gender identity was socially recognized from early childhood within Maros society. Occasional deviations, representing fewer than 10% of cases, may reflect errors in documenting body orientation and position, biological sex estimation, or socially meaningful exceptions. Overall, the obtained results highlight the expression of binary gender division from the youngest age, providing one of the most comprehensive datasets for non-adult individuals.

**Supplementary information:**

The online version contains supplementary material available at https://doi.org/10.1007/s12520-026-02578-3.

## Introduction

In archaeological contexts, sex is generally understood as an estimate of the biological attributes of skeletal remains, whereas gender is interpreted as a social construct (Armelagos 1998:1). A set of biological factors such as chromosomes and hormones produces secondary sexual characteristics that can be observed in the individual skeletal remains and are used for estimating the biological sex in forensic and anthropological contexts (Garofalo and Garvin 2020; Rebay-Salisbury 2024). The manifestation of gender in archaeological assemblages of the Bronze Age is usually visible through iconographic depictions, figurines, rock art, and other visual representations, as well as tools and weapons, dress elements, and ornaments (Sørensen 2000, 2013; Horn and Kristiansen 2018; Harding 2021). Although the study of gender in archaeology today draws on various sources, scholarly discussions are most often centered on funerary rituals. The combination of bodies, material culture, and traces of their funerary treatment offers a unique opportunity to examine the interplay between individuals and their representations in death (Arnold 2008). Grave goods and the body orientation of the buried individuals often reflect both the social status and gender of the deceased, and together with analyses of the skeletal remains from Bronze Age graves, they can offer valuable insights into the relationship between biological sex and gender representation.

### Gender in the Bronze Age burials

Gendered differentiation in burial rites between men and women, particularly in terms of body orientation, position, and grave goods, is evident from the Late Neolithic through the Copper and Early Bronze Age in Europe (Primas 2008; Häusler 2012; Rebay-Salisbury et al. 2022). The estimation of biological sex from skeletal morphology in adult remains has enabled the identification of these distinct mortuary patterns between men and women, as well as the recognition of normative gendered expressions in burial rites. These normative differences between males and females, together with the remains of material culture and iconography, have led authors such as Robb and Harris (2018) to hypothesize that there is a difference in the way gender is constructed between the Neolithic and the Bronze Age in Europe. The authors argue that Neolithic gender was fundamentally different from the binary and stable gender identities that emerged in the Bronze Age. They propose a model of “contextual gender” for the Neolithic, suggesting that gender was not a stable, binary identity but rather situational, fluid, and relevant to specific contexts relative to lifelong personal identity. The Bronze Age, in contrast, witnessed the emergence of more rigid, binary gender systems, closely tied to stable identities and consistently expressed through burial practices, material culture, and iconography (Robb and Harris 2018).

This model of a strong dichotomy between Neolithic and Bronze Age gender construction gave rise to heated debates and critique. Bisserka Gaydarska and colleagues (2023) argue that this oversimplification of gender division overlooks the diversity and complexity of gender roles and identities throughout prehistory. Drawing on diverse regional case studies from Mesolithic and Neolithic burials to Bronze Age mortuary practices and figurine analyses, the authors demonstrate that gender in prehistory was neither uniform nor static, but varied across time, space, age, and social context. Rather than representing a singular transition towards binary identities, the evidence suggests a complex, multi-layered system in which biological sex, age, kinship, and status intersect to shape gender expression. Gaydarska and colleagues (2023) argue for a more nuanced understanding of gender as relational and situational, simultaneously contextual and structured, and warn against essentialist or overly generalized interpretations. Ultimately, the authors highlight that diversity and fluidity, not stability or universality, characterized gender in prehistoric Europe, underscoring the need for intersectional, context-sensitive approaches that integrate bioarchaeological, material, and theoretical perspectives (Gaydarska et al. 2023).

To test the binary gender model in prehistoric burials, Eleonore Pape and Nicola Ialongo (2023) analysed data from seven burial sites spanning the Early Neolithic to the Late Bronze Age in Central Europe. The primary aim of the research was to determine whether the prehistoric gender adhered strictly to a binary model or if non-binary gender variants existed, by bridging the gap between theoretical approaches to gender and empirical archaeological evidence. For this purpose, they compared biological sex estimated from skeletal morphology and assigned gender based on the grave goods of 1252 individuals. The results show that the binary model accounts for most of the sample variability but does not cover all cases. Around 90% of the analysed burials showed matching sex and gender indicators, but 10% presented non-binary or contradictory sex/gender determination (Pape and Ialongo 2023). This study shows that while the binary correspondence between biological sex and gendered representation predominates in prehistoric Central European burials, there are burials that diverge from this pattern. Rather than interpreting these as methodological errors or isolated cases, the authors argue that those burials reflect a small but socially acknowledged non-binary minority. These individuals received standard mortuary treatment, suggesting that gender variance was integrated within the normative social framework of these communities (Pape and Ialongo 2023). Their work highlights the importance of recognizing minority gender expressions in prehistory, viewing them not as exceptions but as integral components of complex social systems of the past. Additionally, the authors further highlight the risk of circular reasoning: errors in determining biological sex can bias how gender is interpreted, thereby reinforcing mistakes in the original sex determinations, underscoring the need for methodological transparency and the integration of biomolecular techniques to reduce bias (Pape and Ialongo 2023).

The study conducted by Rebay-Salisbury and colleagues (2025) addressed these minorities from two Early Bronze Age cemeteries, Franzhausen I and Gemeinlebarn F, in Lower Austria. The authors focused on individuals whose archaeological gender expression (including body orientation, position, and grave goods) contradicted the osteological sex estimation of their skeletal remains. They applied peptide-based sex identification to 34 individuals, showing discrepancies between osteological sex and burial gender expression. The results indicate that most mismatches stem from errors in morphological sex estimation or transcription, demonstrating that the Early Bronze Age burial system in this region exhibits a strong alignment between biological sex and gendered burial treatment. Nevertheless, a few exceptions remain that can be attributed to error, post-depositional disturbance, or mixed-message mortuary practices (Rebay-Salisbury et al. 2025).

However, due to methodological challenges in determining the biological sex of immature skeletal remains (Schutkowski 1993; Lewis 2006; Cunningham et al. 2016), combined with a historical lack of interest in the youngest members of prehistoric communities, the gender roles of children in the Bronze Age remained unknown for the longest time. Indeed, the archaeology of childhood is as “young” as the archaeology of gender (Murphy 2017; Crawford et al. 2018; Baxter 2022), and the limitations of osteological sex estimation have left us largely without empirical data, forcing us to rely on assumptions. Children’s burials, when following similar sex-specific rituals, are most often assumed to mirror those of adults (Neugebauer 1991; Häusler 2012). Recent advances in genetic sex determination have begun to provide new insights into the lives and deaths of Bronze Age boys and girls. For the Early Bronze Age cemetery of Franzhausen I, Austria, a study conducted by Rebay-Salisbury and colleagues (2022) based on sex-specific amelogenin fragments in dental enamel has shown that children’s burials follow the same sex-specific norms as adults, notably regarding body position and associated grave goods. This research has demonstrated that from the earliest age, boys were buried in the same manner as adult men and girls in the same manner as adult women (Rebay-Salisbury et al. 2022).

### Research aim

This study utilizes the identification of sex-specific peptides in dental enamel to determine the biological sex of non-adults aged from 9 to 12 months to 18–22 years, buried at two Bronze Age sites in Mokrin and Ostojićevo. The results of the sex identification are then employed to assess whether children were assigned a binary gender identity that is reflected in the funerary treatment that follows the treatment of adults, i.e., whether boys follow the funeral ritual typically practiced for adult men, and girls the one for adult women, in terms of the body position and orientation.

## Material

**Fig. 1:**
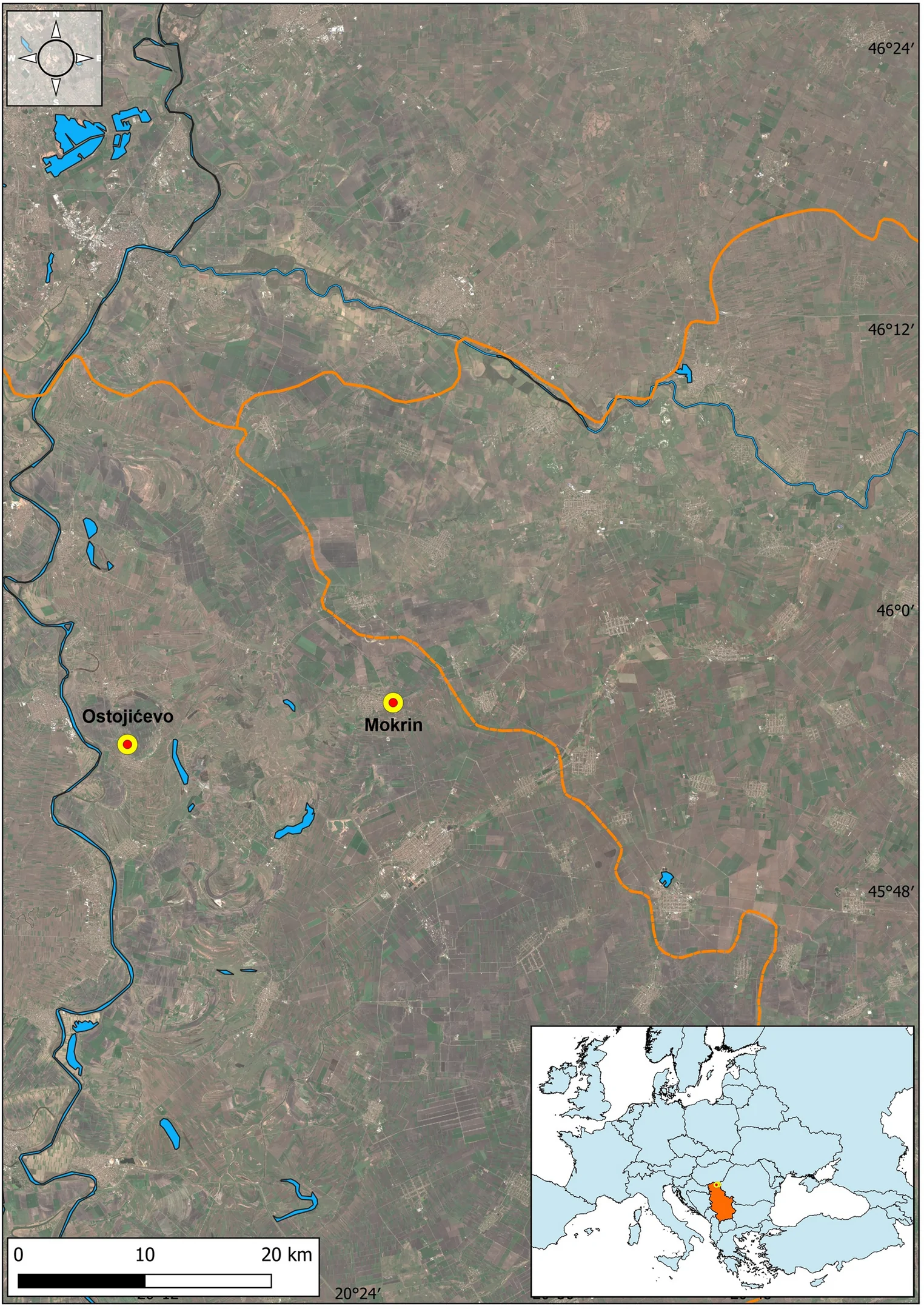
Map with Mokrin and Ostojićevo sites, Banat, northern Serbia, author: Jugoslav Pendić

For this research, we have analysed data on total of 128 non-adult individuals from two Bronze Age sites, Mokrin and Ostojićevo (Fig. 1), while our study focuses on 77 non-adult individuals with available biological sex determination. Both sites are associated with the Maros culture, which extended across present-day Hungary, Romania, and northern Serbia (Garašanin 1983). Chronologically, the Maros culture is defined within the Early and Middle Bronze Age (Girić 1971), with a series of radiocarbon dates placing its duration between 2700 and 1700/1500 cal. BC, and its peak around 2000 BC (O’Shea 1992, 1996; O’Shea et al. 2019; Milašinović et al. 2024; Krečković Gavrilović et al. 2025). The Maros culture is well known for its normative funerary ritual, structured around the biological sex of the deceased. Burials in Maros cemeteries were predominantly individual inhumations, with individuals interred in a flexed position, laying on either the right or left side. The orientation of the bodies as well as the graves, including both the pit and the body’s positioning, were aligned along a north-south or south-north axis, depending on the sex of the deceased (O’Shea 1996). This norm in the funerary ritual is noted based on the adult burials at multiple Maros sites, where it was possible to estimate sex based on the skeletal remains and by analysing attributes of the burials like grave goods, depth, and shape of the burial pit (O’Shea 1996; Krečković Gavrilović et al. in press).

### Mokrin necropolis

The Mokrin necropolis is among the most extensively studied and published sites of the Maros culture, situated on the eastern outskirts of the village of Mokrin near Kikinda in northern Banat, Serbia (Fig. 1; Girić 1971). Initial excavations carried out in the 1960 s uncovered 312 graves (Girić 1971), while more recent investigations conducted between 2020 and 2024 yielded an additional seven burials (Krečković Gavrilović et al. 2025). The cemetery was used between 2100 and 1800 BC (O’Shea 1992; Krečković Gavrilović et al. 2025). Both adult and non-adult individuals were primarily buried in individual graves, with a few exceptions, including three double burials, one grave containing three individuals, and nine cremations or symbolic burials. Burial pits at Mokrin were typically rectangular with rounded corners, with the longer axis aligned in a north-south or south-north direction, mirroring the orientation of the interred individuals. Less commonly, burial pits exhibited square, circular, or ellipsoid base shapes. The dimensions of the pits varied according to the body size of the individual (Girić 1971). The deceased were almost universally interred in a flexed position, lying on either the left or right side, with arms bent at the elbow, upper arms drawn toward the chest, and legs flexed at the knees. The face was typically oriented towards the east (Girić 1971). A defining characteristic of the Maros culture’s funerary ritual is the role of biological sex in determining burial orientation (O’Shea 1996). Males were predominantly placed on their left side, with their head facing north and their face turned toward east, resulting in a grave and body orientation along a north-south axis. In contrast, females were interred on their right side, with their head facing south and their face turned toward the east, resulting in a south-north body and grave orientation (Girić 1971), without spatial differentiation observed between the burials of adult males and females (Girić 1971; O’Shea 1996). Apart from the orientation, certain grave goods also show correlations with the individuals’ biological sex: weapons are characteristic of males, whereas females usually have some kind of jewelry, such as bone needles, metal needles, or necklaces (O’Shea 1996; Krečković Gavrilović et al. in press). Also, it should be noted that certain classes of grave goods (daggers, axes, sashes, head ornaments, bone needles) in adult burials exhibit a correlation with the social status of the deceased (O’Shea 1996).

Compared to adult burials, the skeletal preservation of non-adult individuals at Mokrin is notably poor. This is primarily due to the burial practices associated with children, as their graves were typically shallower (Girić 1971; Amzirkov 2020), making them more susceptible to post-depositional disturbances, including agricultural activity. Additionally, taphonomic processes, soil composition, and the biological characteristics of non-adult remains, such as incomplete ossification and disease-related fragility, further contributed to their poor preservation. Despite these challenges, in cases where the body position and orientation of non-adult skeletons could be determined, they generally adhered to the same funerary norms as adults, with a north-south or south-north orientation, placed on their left or right side, and the face turned eastward (except in the case of grave 193; Girić 1971). Notably, no burials of children under 2 years were identified at the Mokrin necropolis (Amzirkov 2020). Evidence from Maros culture settlements suggests infants were sometimes interred within settlements rather than in formal cemeteries (Rega 1997; O’Shea 1996). However, the number of documented infant burials in settlements remains significantly lower than the expected number of babies based on demographic estimations of the Maros population. This suggests that settlements were not the sole location for infant burials, and alternative burial practices may have existed (O’Shea 1996: 147), such as water or sky burials.

### Ostojićevo necropolis

The necropolis of Ostojićevo is a lesser-known but equally important site, with 285 discovered graves, out of which 78 belong to the Early Bronze Age Maros horizon wich will be analysed in this study and 207 to the Middle Bronze Age horizon (Girić 1995; Милашиновић 2008; Marin et al. 2024). Like Mokrin, Ostojićevo is located near Kikinda in northern Banat, Serbia, on the outskirts of the eponymous village (Fig. 1). It is situated along the bank of a former meander of the Tisa River. The site was first recorded in 1954 during canal embankment works and later systematically excavated between 1981 and 1991 under the National Museum in Kikinda (Girić 1959, 1995; Милашиновић 2008). The absolute chronology of this site indicates that Ostojićevo was founded several centuries after the Mokrin necropolis, around 1900 cal BC, and remained in use until ca. 1550 cal BC (O’Shea et al. 2019; Milašinović et al. 2024;

Burial practices for the Early Bronze Age graves at Ostojićevo followed the characteristic norms of the Maros culture: bodies were typically laid in a flexed position, oriented along a north-south axis, and facing east, without differences in spatial distribution between burials of adult males and females (Милашиновић 2008). A small number of individuals, however, deviated from this pattern, being aligned northeast, northwest, southeast, or southwest, while some showed more pronounced east-west orientations (Милашиновић 2008). The graves are most frequently rectangular with rounded corners, while ellipsoid and square forms occur less commonly. In several cases, traces of organic material were noted at the base, possibly representing remnants of a leather lining of the grave or cover of the deceased (Милашиновић 2008). The burials at Ostojićevo exhibit patterns similar to those observed in Mokrin, particularly in terms of the association of individual classes of grave goods with the sex and social status of the buried individual. The only weapon was found with a male individual, whereas bone and metal pins occur exclusively in female graves. However, none of these associations reached statistical significance, likely due to the small sample size (Krečković Gavrilović et al. in press). Children buried at Ostojićevo generally adhered to the same funerary norms as adults, with a north-south or south-north orientation, and were placed on their left or right side, facing east (except for graves 214 and 250; Милашиновић 2008).

### Sample

The criteria for including individuals in this study were based on the preserved dentition, and the age was limited to 18 to 22 years, as their growth and development was not completed. At the Mokrin necropolis, 104 burials were classified as non-adults, but the skeletal remains were available for only 67, 59 of which had preserved dentition for peptide analysis. One more individual was added to the Mokrin sample, as the genetic sex was determined in a previous aDNA study (Žegarac et al. 2021). The sample from the earlier Maros phase of Ostojićevo is smaller. Twenty-four remains were classified as non-adult, of which the skeletal remains were preserved for 22 individuals. The dentition for peptide analysis was available for 17 of these individuals. Overall 77 individuals of known biological sex and of available data for orientation and body position were included in this study (SI1).

## Methods

We have conducted a standard anthropological analysis and peptide-based sex identification for all observed individuals where dentition was available. We have compared the obtained results with available data on body orientation and position in the grave taken from the previously published literature (Girić 1971; Милашиновић 2008) and for one grave (no. 314) from field documentation. Estimates of age at death were obtained using standard anthropological methods, based on the degree of dental eruption of both deciduous and permanent teeth (Ubelaker 1978), long bone lengths (Fazekas and Kósa 1978; Stloukal and Hanáková 1978; Scheuer and Black 2000), and the stage of epiphyseal fusion (Ferembach et al. 1980). Depending on the preservation of the skeletal remains and stage of development, different methods were used accordingly. All measurements were taken according to the standards proposed by Buikstra and Ubelaker (1994). As the body orientation and position of the individual are very distinctive in the Maros funerary ritual, we have compared whether the assigned male or female body orientation and position noted in adult burials is the same in children’s burials. We have also compared the gendered body position and orientation in children’s burials with the biological sex obtained via peptide-based sex identification. In other words, we have examined whether boys have the same body position and orientation as adult men, and whether girls have the same body position and orientation as adult women. To test weather body orientation was associated with sex; we constructed a contingency table and assessed the statistical significance of the association using the Freeman and Haltons’s (1951) extension of Fisher’s exact significance test for contingency tables implemented in IBM SPSS Version 23. The strength of the associations was measured using Cramer’s V, also implemented in IBM SPSS Version 23.

### Peptide-based sex identification

The analysis of sexually dimorphic amelogenin fragments in human dental enamel by mass spectrometry has become the gold standard for sex estimation in anthropology and archaeology (Stewart et al. 2017; Parker et al. 2019; Buonasera et al. 2020; Madupe et al. 2025). This minimally invasive method is particularly useful for sub-adult individuals (Gowland et al. 2021; Rebay-Salisbury et al. 2022), intermingled and incompletely preserved skeletons (Cintas-Peña et al. 2025; García Sanjuán et al. 2026), and ambiguous morphological cases (Rebay-Salisbury et al. 2025).

Peptides were extracted following our peptide extraction protocol (see Rebay-Salisbury et al. 2025), adapted from (Stewart et al. 2017), and identified by ultra-high-performance liquid chromatography-high-resolution mass spectrometry (UHPLC-HRMS). The sex of males was inferred by the presence of Amelogenin X and Y, the sex of females by Amelogenin X without Y. All samples met the quality control threshold. The raw mass spectrometric data were uploaded to the MassIVE repository under the accession number MSV000100774.

## Results

### Mokrin

A total of 60 burials were analysed; one case was excluded due to missing data, leaving 59 valid cases for statistical analysis. The Fisher’s exact test was performed to explore the association between biological sex and body orientation. The result revealed a statistically significant relationship between these two variables (*p* < 0.001). We calculated Cramer’s V as a measure of the strength of the effect. The value of Cramer’s V = 0.80 (*p* < 0.001) indicates a strong association between biological sex and body orientation, with a high degree of predictability of individual sex based on body orientation.

Regarding the body position and the peptide sex correlation, all 60 cases were included in the analysis. The Fisher’s exact test suggested a significant association between peptide-determined sex and body position (*p* < 0.001). The value of Cramer’s V is 0.56, which indicates a moderate, but statistically significant, association between biological sex and body position (probably due to the high percentage of individuals for whom the body position could not be determined).

**Fig. 2:**
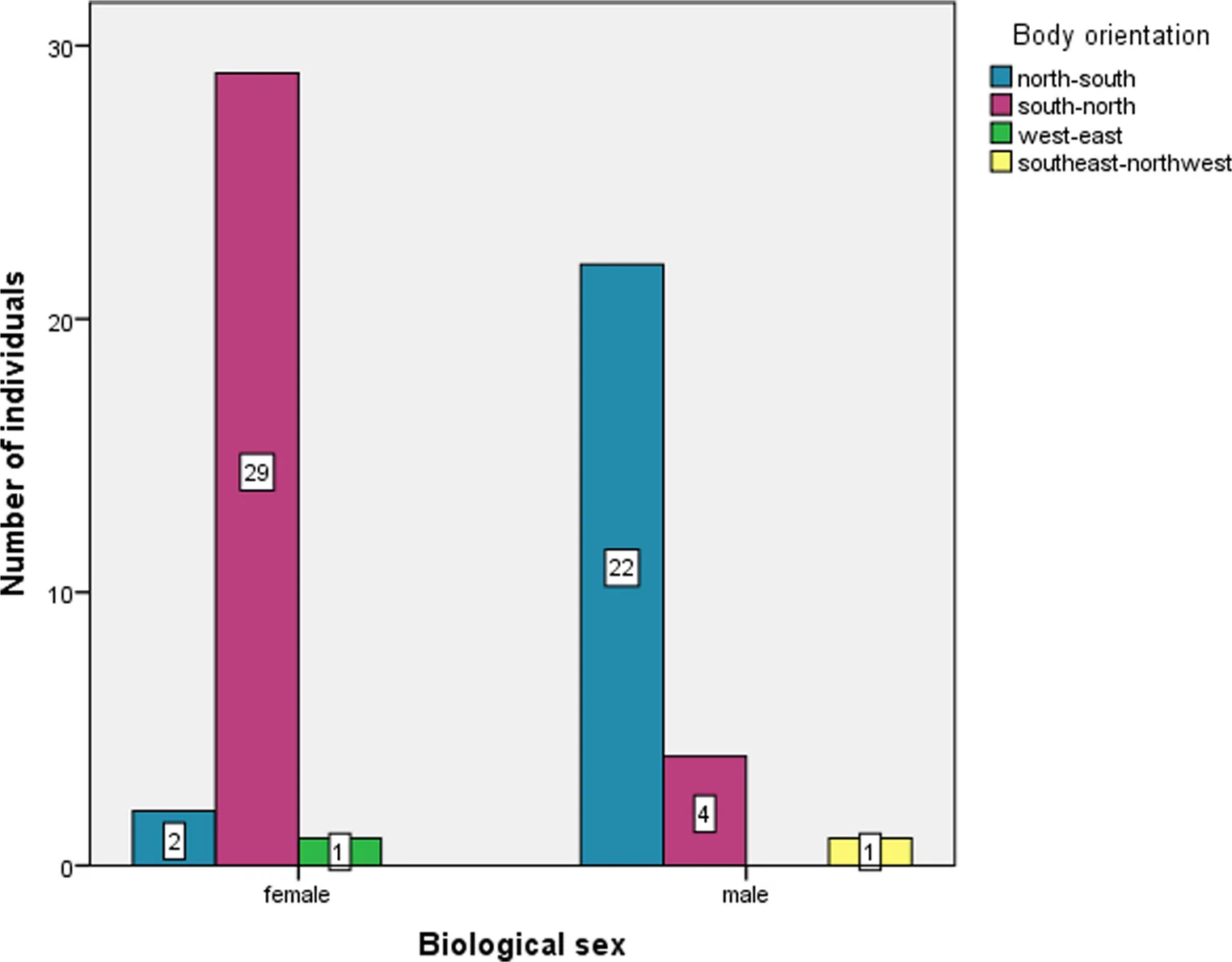
Body orientation and biological sex association at Mokrin necropolis

**Fig. 3:**
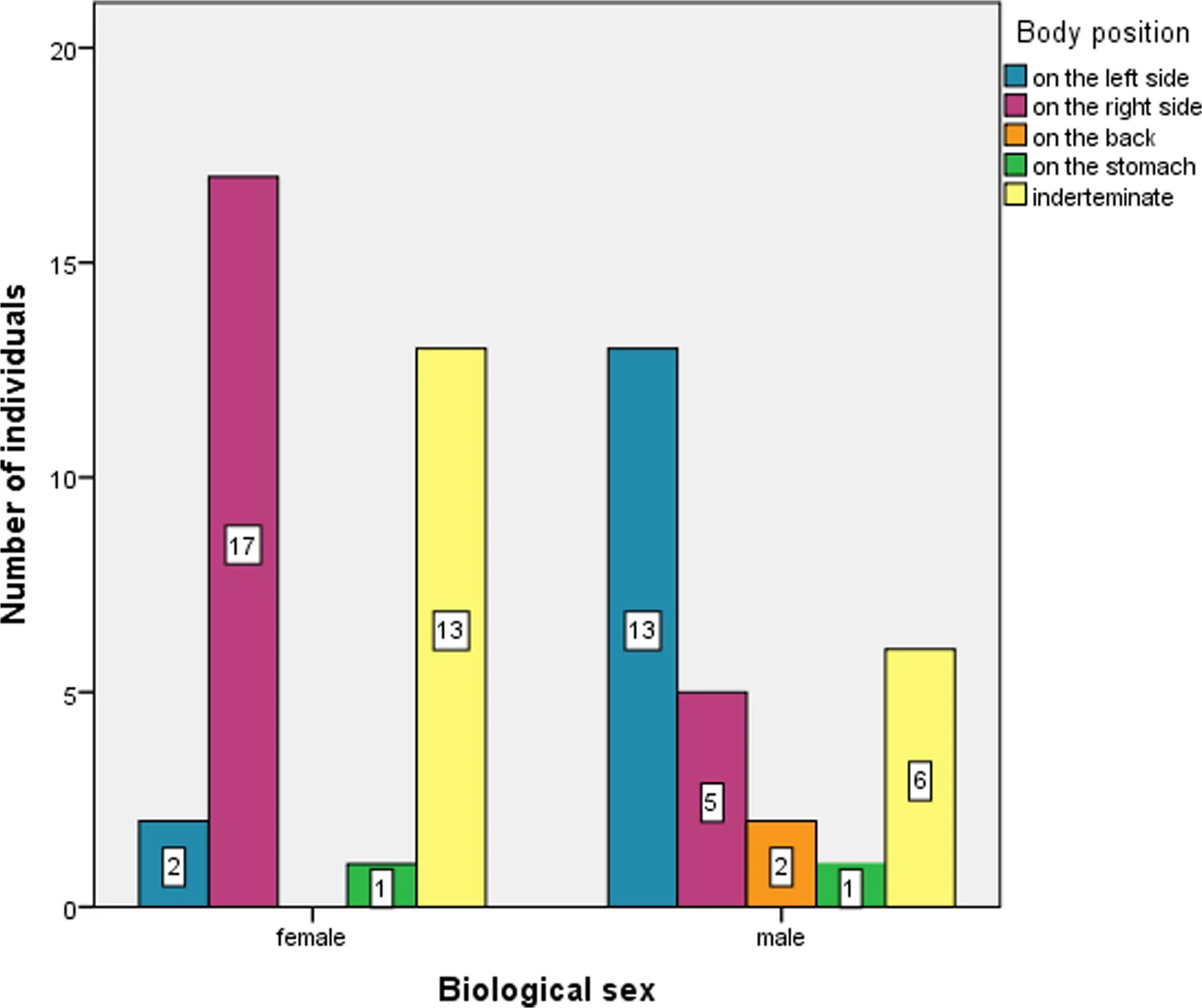
Body position and biological sex association at Mokrin necropolis

Taken together, these results provide evidence that chromosomal sex is strongly associated with burial orientation and moderately associated with the side on which the body is placed in a grave at the Mokrin necropolis. The overwhelming predominance of girls oriented south-north, placed on their right side, and boys oriented north-south, placed on their left side, reflects a clear cultural practice rather than a random distribution of burial positions (Figs. 2 and 3).

### Ostojićevo

From the Ostojićevo necropolis 16 individuals had both peptide-based sex determinations and body orientations documented. The results revealed a statistically significant relationship between biological sex and body orientation (*p* < 0.001). The value of Cramer’s V = 1 (*p* < 0.001) indicates a strong and significant association between biological sex and body orientation. 

As for the analysis of the association between peptide-determined sex and the body position, seventeen individuals from Ostojićevo were analysed. The Fisher’s exact test suggested association between biological sex and the body position is statistically significant (*p* < 0.001). Notably, no males were placed on the right side, and no females on the left Effect size calculations showed a very strong relationship, with Cramer’s V value of 1, with an associated p value < 0.001. This suggests that biological sex was an almost perfect predictor of the side on which the body would be positioned during burial at Ostojićevo necropolis.

**Fig. 4:**
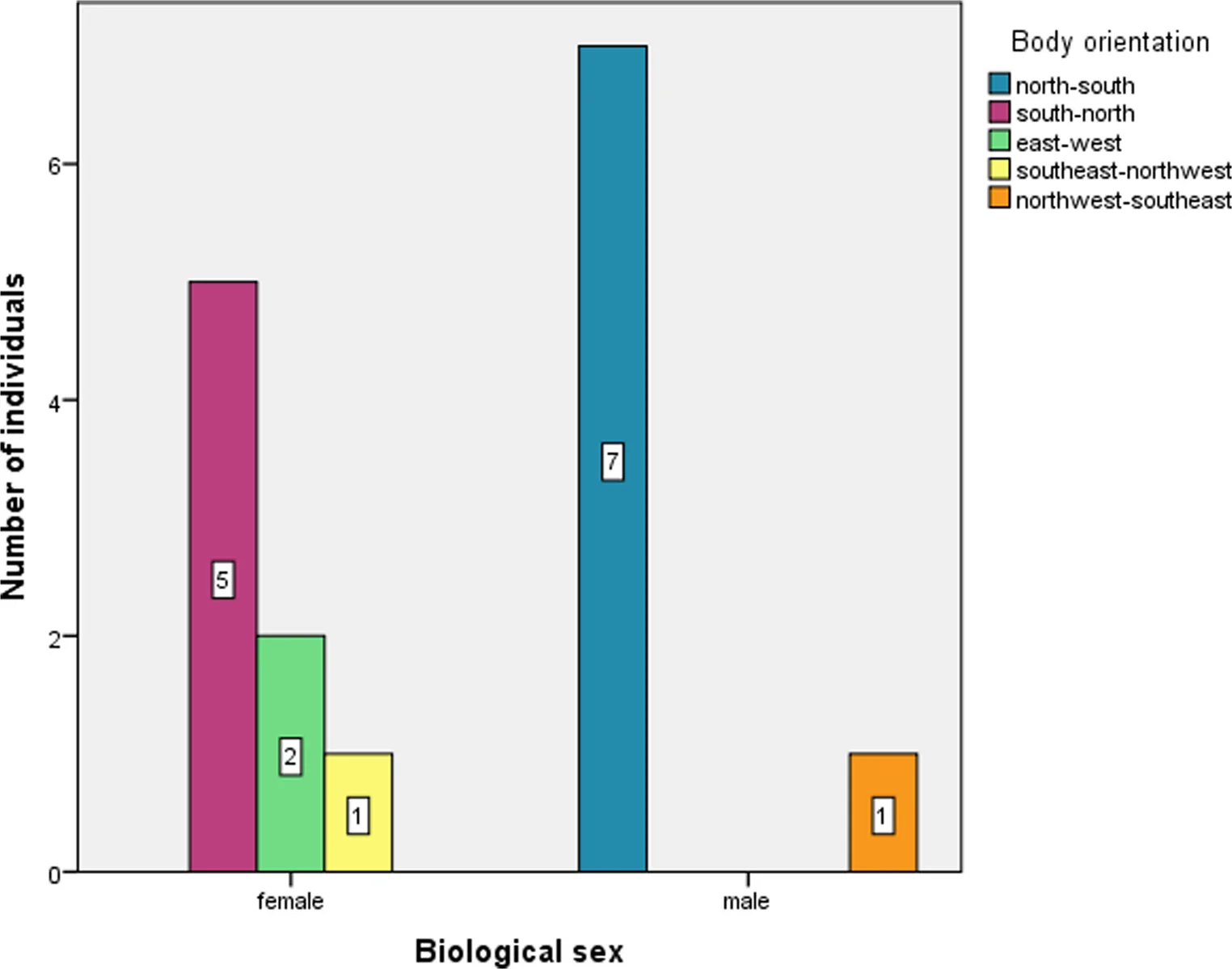
Body orientation and biological sex association at Ostojićevo necropolis

**Fig. 5:**
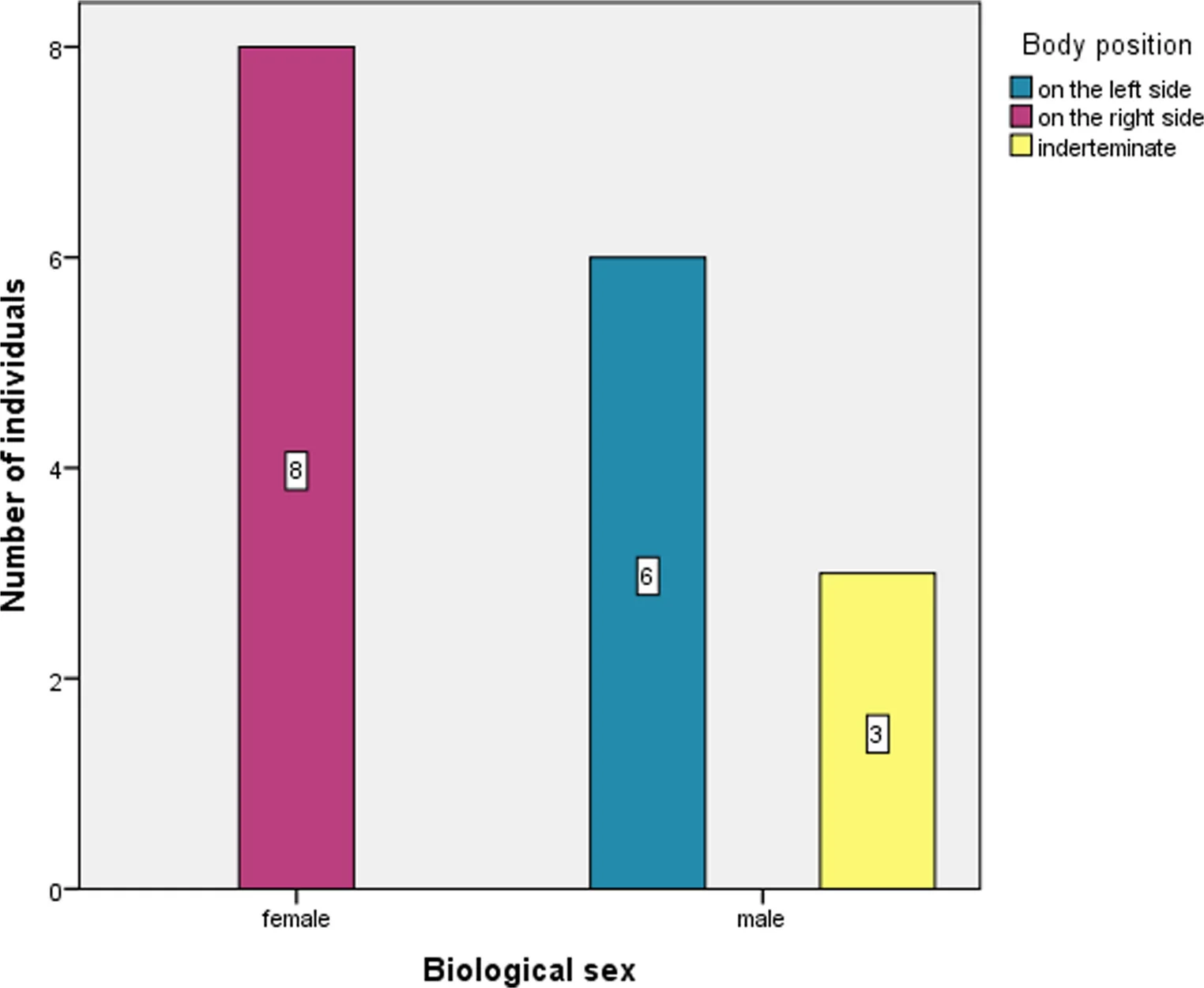
Body position and biological sex association at Ostojićevo necropolis

These results demonstrate that at the Ostojićevo necropolis, orientation and body position were not random but followed clear sex-based funeral norms, with girls preferentially interred along a south–north axis on their right side, and boys along a north–south axis on their left side, consistent with Maros culture burial customs (Figs. 4 and 5).

## Discussion

The results from both sites, Mokrin and Ostojićevo, demonstrate that body positioning was closely determined by biological sex for two key dimensions of burial practice, cardinal orientation and side of the body. At the Mokrin necropolis, girls were overwhelmingly interred on the right side and generally oriented along a south–north axis, whereas boys were more commonly placed on the left side with a north–south orientation. Although some variation occurred, particularly in minor alternative orientations (e.g., east–west, southeast–northwest), Statistical tests confirmed that both body position and orientation were significantly associated with sex. The Ostojićevo necropolis replicated these findings in a smaller but even clearer dataset. Here, girls were almost exclusively placed on the right side and most often oriented south–north, while boys were predominantly placed on the left side with a strongly consistent north–south orientation. Both relationships were statistically significant, and the strength of association was particularly pronounced for the body position, approaching a perfect correlation. These results are consistent with previous studies conducted on adult samples from both sites, where biological sex was estimated anthropologically from skeletal remains (O’Shea 1996; Krečković Gavrilović et al. in press). They also confirm that children, from the earliest ages onward, were buried according to the same practices as adults, except for those younger than one year.

Still, on Mokrin necropolis there are graves where the biological sex does not match the burial orientation, both among adults and children. For most adult burials with non-matching orientation (Порчић 2010; Matić 2012) and available dentition for peptide sex determination, the results have shown that the estimation of biological sex based on skeletal remains was incorrect (Krečković Gavrilović et al. in press). Among the non-adult sample, there are six exceptions (Table 1). In graves 25, 103, and 236 at Mokrin, peptide-based sex identification revealed that boys were buried according to the female norm, with their heads facing south. The same applies to grave 220, where sex was determined by ancient DNA analysis (Žegarac et al. 2021). None of the burials includes any gender-typical grave goods.

**Table 1:** Exceptions from the norm

Site	Grave No.	Body orientation	Body position	Biological sex	Age	Grave goods
Mokrin	**25**	south-north	right	boy	6–7 years	pottery
Mokrin	**103**	south-north	right	boy	4 years	pottery
Mokrin	**220**	south-north	right	boy*	10–11 years	without grave goods
Mokrin	**236**	south-north	right	boy	7 years	pottery
Mokrin	**216**	north-south	left	girl	8–9 years	without grave goods
Mokrin	**238**	north-south	left**	girl	14–15 years	pottery, animal bones

*sex was determined with aDNA analysis (Žegarac et al. 2021)

**Fig. 6:**
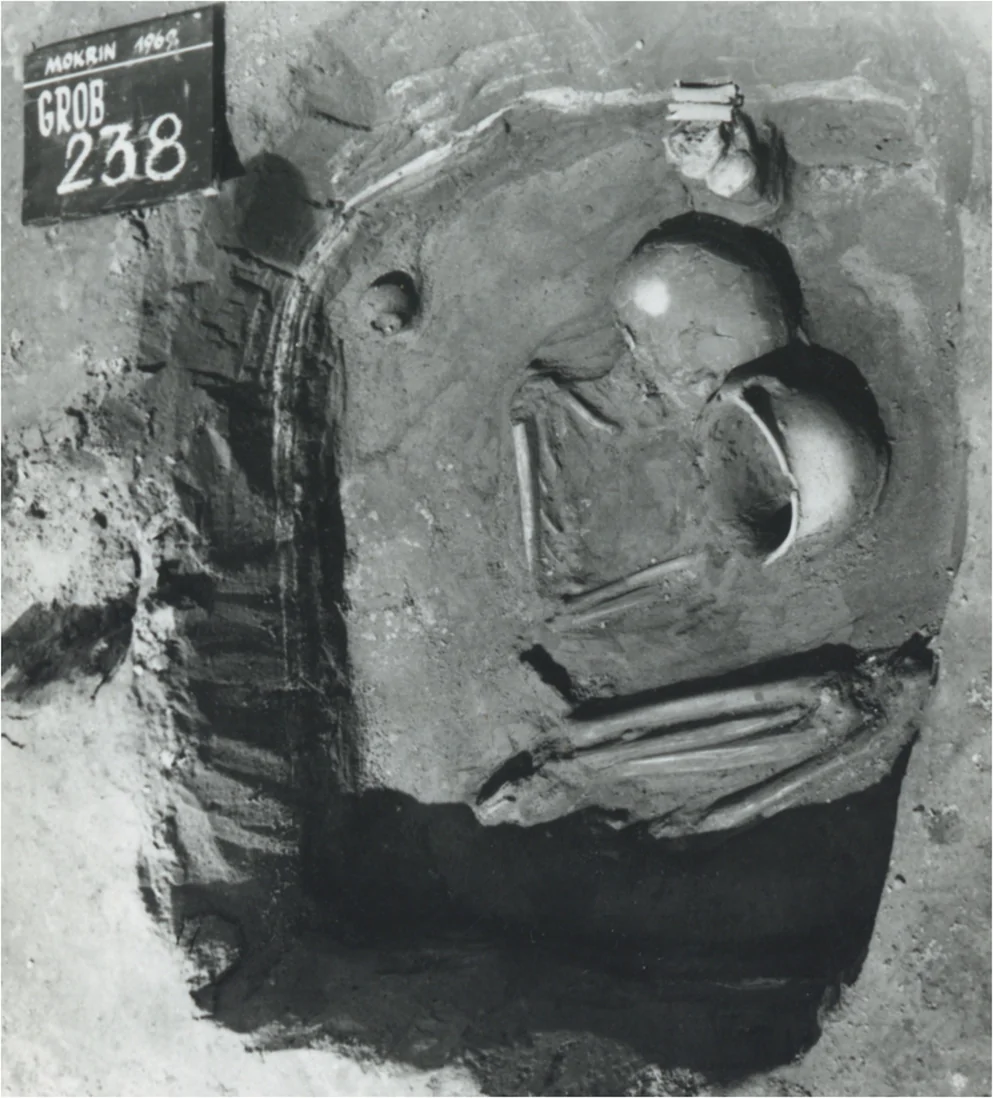
Grave 238, girl buried on her left side in North-South orientation. Field documentation from the National Museum in Kikinda, Serbia

** In the published monograph (Girić 1971), it is written on the right side, but by checking the photo documentation available from the National Museum in Kikinda, it was visible that the individual was buried on their left side (Fig. 6)

In contrast, Mokrin graves 216 and 238 contain girls buried in the male norm with their heads to the north. What is notable is that the body position matches the peptide-determined sex perfectly. In these cases, we must consider the amount of preserved skeletal remains recorded during the excavation, which may lead to a possible incorrect orientation. This may explain the situation in Graves 25, 103, and 236, where, in the field photo documentation, we can see poor preservation of the remains. However, remains were well-preserved in Graves 220, 216, and 238. These graves show no specific distinguishing features; they vary in age, position within the necropolis, and grave goods. They do not follow any clear pattern, except that they are among the burials less equipped with grave goods. However, it should be noted that children’s burials generally lack grave goods compared to adult burials (Amzirkov 2020).

A similar situation was observed at the Bronze Age cemetery Franzhausen I in Austria, where peptide-based sex identification was performed on 75 individuals aged between 0 and 12 years old. The concordance between the archaeological gender classification (body orientation, position, and grave goods) and peptide-based sexing was very high (62 of 63 individuals, 98.4%). This remarkably high correlation suggests that the Bronze Age community of Franzhausen I recognized the biological sex of children and buried them accordingly, with one exception – a 4–5-year-old female buried in male position (Rebay-Salisbury et al. 2022). Similarly, for most of the adult individuals not conforming to the highly gendered burial pattern from the cemeteries of Franzhausen I and Gemeinlebarn F, it was shown that there was an error in the documentation or the osteological sex estimation (Rebay-Salisbury et al. 2025).

## Conclusion

Taken together, the results from the two necropolises indicate that children’s burial practices on Mokrin and Ostojićevo followed clear, binary sex-based cultural prescriptions, the same as for adults. The consistency across different communities within the Maros cultural sphere suggests a shared mortuary code, in which females were symbolically aligned with the right side and a south–north orientation, and males with the left side and a north–south orientation was applied from the earliest age. Minor deviations, such as occasional east–west orientations or indeterminate body positions, including opposite orientation, may reflect local variation, post-depositional distortion, or individual exceptions. However, they do not obscure the overarching rule. These few exceptions to the dominant pattern of gendered burial highlight the possibility that additional social or cultural factors, or other extenuating circumstances (e.g., simple error or convenience), may influence burial practices. Still, the existing pattern demonstrates that gender roles in the Maros society were materially expressed in mortuary practice, reinforcing binary gender distinctions through standardized bodily placement from the earliest age. The replication of the rule set across multiple cemeteries strengthens the interpretation that these practices were culturally codified and widely recognized within the Maros group.

## Supplementary information

Below is the link to the electronic supplementary material.

Supplementary File 1 (XLSX 42.4 KB)

## Data Availability

All the mass spectrometry proteomics data have been deposited to the MassIVE repository, and the age and burial data in provided in Supplementary (SI1) material.
